# Trans-Sodium Crocetinate Ameliorates High-Altitude Acute Lung Injury via Modulating EGFR/PI3K/AKT/NF-κB Signaling Axis

**DOI:** 10.3390/nu17152406

**Published:** 2025-07-23

**Authors:** Keke Liang, Yanlin Ta, Liang Xu, Shuhe Ma, Renjie Wang, Chenrong Xiao, Yue Gao, Maoxing Li

**Affiliations:** 1 College of Pharmacy, Gansu University of Chinese Medicine, Lanzhou 730013, China; 18394248206@163.com (K.L.); tyl20250510@163.com (Y.T.); xuliang021026@163.com (L.X.); mclsxka@163.com (S.M.); wangrenjiegy@163.com (R.W.); 2 Department of Pharmaceutical Sciences, Beijing Institute of Radiation Medicine, Beijing 100850, China; xiaocr@sina.com

**Keywords:** high-altitude acute lung injury, trans-sodium crocetinate, EGFR/PI3K/AKT/NF-κB signaling pathway, network pharmacology, molecular dynamics simulation

## Abstract

Objectives: *Saffron*, a traditional Chinese medicine, is renowned for its pharmacological effects in promoting blood circulation, resolving blood stasis, regulating menstruation, detoxification, and alleviating mental disturbances. Trans-crocetin, its principal bioactive component, exhibits significant anti-hypoxic activity. The clinical development and therapeutic efficacy of trans-crocetin are limited by its instability, poor solubility, and low bioavailability. Conversion of trans-crocetin into trans-sodium crocetinate (TSC) enhances its solubility, stability, and bioavailability, thereby amplifying its anti-hypoxic potential. Methods: This study integrates network pharmacology with in vivo and in vitro validation to elucidate the molecular targets and mechanisms underlying TSC’s therapeutic effects against high-altitude acute lung injury (HALI), aiming to identify novel treatment strategies. Results: TSC effectively reversed hypoxia-induced biochemical abnormalities, ameliorated lung histopathological damage, and suppressed systemic inflammation and oxidative stress in HALI rats. In vitro, TSC mitigated CoCl_2_-induced hypoxia injury in human pulmonary microvascular endothelial cells (HPMECs) by reducing inflammatory cytokines, oxidative stress, and ROS accumulation while restoring mitochondrial membrane potential. Network pharmacology and pathway analysis revealed that TSC primarily targets the EGFR/PI3K/AKT/NF-κB signaling axis. Molecular docking and dynamics simulations demonstrated stable binding interactions between TSC and key components of this pathway. ELISA and RT-qPCR confirmed that TSC significantly downregulated the expression of EGFR, PI3K, AKT, NF-κB, and their associated mRNAs. Conclusions: TSC alleviates high-altitude hypoxia-induced lung injury by inhibiting the EGFR/PI3K/AKT/NF-κB signaling pathway, thereby attenuating inflammatory responses, oxidative stress, and restoring mitochondrial function. These findings highlight TSC as a promising therapeutic agent for HALI.

## 1. Introduction

Regions at high altitudes, typically identified as those situated above 2500 m, exhibit characteristics such as reduced atmospheric pressure, low oxygen contents, cold climates, and high radiation exposure. Of these elements, low oxygen availability poses a significant challenge for people who are traveling to, residing in, or working in elevated environments [1]. High-altitude acute lung injury (HALI) refers to a lung condition stemming from hypoxia experienced by individuals who ascend quickly to elevated elevations, showing symptoms such as pulmonary inflammation, fluid accumulation, and damage to the alveolar/capillary interface [2]. In clinical settings, this condition is exhibited through respiratory failure syndrome and worsening hypoxemia, potentially advancing to acute respiratory distress syndrome (ARDS) [3]. HALI has a high mortality rate, and its pathogenesis remains unclear, making targeted treatment difficult. Current therapies include oxygen supplementation, rapid descent, and pharmacological interventions such as carbonic anhydrase inhibitors (e.g., acetazolamide), glucocorticoids (e.g., dexamethasone), and β2-adrenergic agonists (e.g., salmeterol) [4]. However, drug resistance, adverse effects, and hypoxia-reoxygenation injury caused by physical therapies exacerbate the risk of HALI. Recently, Traditional Chinese Medicines (TCMs) and their bioactive components have shown promise in treating HALI due to their “multi-target, multi-pathway” mechanisms, which align with the complex pathophysiology of HALI.

In TCM theory, HALI is categorized as “Qi deficiency and blood stasis” (Qi Xu Xue Yu), where disrupted Qi circulation leads to blood stagnation and the pathological state of “phlegm-stasis interaction” (Tan Yu Hu Jie), consistent with modern mechanisms such as inflammatory infiltration, oxidative stress, and apoptosis. However, current TCMs for HALI, including *Rhodiola*, *Ginseng*, and *Astragalus*, face limitations such as high cost, unclear pharmacological mechanisms, or limited availability [5]. Thus, developing low-toxicity, high-efficacy anti-hypoxic agents is crucial for improving health outcomes in high-altitude populations.

*Saffron* is a perennial herb belonging to the Iridaceae family. According to the 2020 edition of the Chinese Pharmacopoeia, it is characterized by its sweet flavor and calming nature and is utilized to address various conditions such as menstrual issues, postpartum blood stasis, warm and toxic hair spots, palpitations, and depression [6]. One of its primary active compounds, crocetin, exhibits several pharmacological properties, including anti-inflammatory, antioxidant, neuroprotective, and anti-tumor effects [7]. Recently, it has demonstrated notable benefits in managing respiratory ailments. In a study conducted by Teng et al. [8], animal experiments indicated that trans-crocetin can considerably alleviate radiation-induced thickening of the alveolar walls and damage to alveolar structure in mice. Additionally, molecular mechanism investigations revealed that this compound effectively mitigates radiation-induced lung injury by downregulating the expression of the TNF receptor superfamily member 10b (*Tnfrsf10b*) gene while inhibiting the pathways associated with necrotic apoptosis. In models of ovalbumin-induced allergic asthma, trans-crocetin has been shown to significantly decrease contents of Th2 inflammatory markers and the expression of oxidative stress indicators while also enhancing airway hyperresponsiveness and lung function parameters [9]. Despite the promising potential of trans-crocetin for treating lung conditions, research focused on high-altitude lung injury (HALI) remains scarce. HALI, a prevalent pulmonary issue in high-altitude regions, encompasses various pathological processes, including hypoxia-induced inflammatory responses, oxidative stress, and cellular apoptosis, which play critical roles in its development [10]. Although the multi-target action characteristics of trans-crocetin acid are theoretically consistent with the complex pathological mechanism of HALI, its clinical application and transformation are limited by the easy oxidative decomposition of conjugated double bonds in its chemical structure and the low bioavailability caused by poor water solubility. Therefore, the sodium salt modification strategy was adopted to prepare sodium trans-saffron (TSC) from saffron acid, and its dissolution stability was improved through ionization modification, providing a solid foundation for subsequent formulation design, pharmacokinetic studies, and preclinical evaluation.

Therefore, this study used network pharmacology techniques to construct a TSC target disease interaction network and screen core targets and key pathways. Further validate the binding mode and stability of TSC with target proteins through molecular dynamics simulations, revealing its structure/activity relationship. Finally, the pharmacological mechanism of TSC was validated by combining animal models and cellular hypoxia models, aiming to provide a theoretical basis for the development of safe and effective anti-high-altitude acute lung injury drugs.

## 2. Materials and Methods

### 2.1. Animals, Cells, Reagents, and Instrumentation

SPF Male KM mice (4–5 weeks old, 20 ± 2 g) and SD rats (6–8 weeks old, 200 ± 20 g) were obtained from Beijing Vital River Laboratory Animal Technology Co., Ltd. (Beijing, China; SCXK (Jing) 2021-0011). The animals were maintained under controlled conditions (20–25 °C, 55 ± 5% humidity). All experimental protocols were approved by the Animal Ethics Committee of the Military Medicine Research Institute (Beijing, China; Approval No. IACUC-DWZX-2023-P595). Human pulmonary microvascular endothelial cells (HPMECs) were acquired from Wuhan Procell Life Technology Co., Ltd. (Wuhan, China).

Trans-sodium crocetinate (TSC, No. nk1-00080) was sourced from Chengdu Nakeli Biotechnology Co., Ltd. (Chengdu, China). Pentobarbital (No. 57-33-0) and heparin sodium (No. 20220527) were purchased from Sigma-Aldrich (St. Louis, MO, USA) and Sinopharm Chemical Reagent Co., Ltd. (Beijing, China), respectively. Acetazolamide (Lot No. RH102875) was provided by Shanghai Yien Chemical Technology Co., Ltd. (Shanghai, China). DGDR-10II dry electrochemical blood gas test strips and kits (Lot Nos. W36418B03H, W45118C34H) were obtained from Guangzhou Wondfo Biotech Co., Ltd. (Guangzhou, China). The JC-1 Mitochondrial Membrane Potential Assay Kit (No. B1012), DCFH-DA ROS Detection Kit (No. C1300-1), and Calcein-AM/PI Double Staining Kit (No. C542) were supplied by Beijing Pulilai Gene Technology Co., Ltd. (Beijing, China) and Dojindo Molecular Technologies, Inc. (Kumamoto, Japan). The CCK-8 assay kit (No. C0005) was procured from TargetMol (Boston, MA, USA). TNF-α (No. 250508250R, 250508285R), MCP-1 (No. 250508264R, 250508290R), IL-10 (No. 250508269R, 250508294R), MDA (No. 250508273R, 250508306R), GSH (No. 250508278R, 250508317R), SOD (No. 250508282R, 250508322R), EGFR (No. 250519124R), PI3K (No. 250519133R), AKT (No. 250519138R), and NF-κB (No. 250519146R) were provided by Jiangsu Meimian Industrial Co., Ltd. (Yancheng, China).

The DYC-9070 hypobaric hypoxia chamber was manufactured by Fenglei Aviation Ordnance Co., Ltd. (Anshun, China). The BGA-102 portable blood gas analyzer and hematology analyzer were provided by Guangzhou Wondfo Biotech Co., Ltd. (Guangzhou, China) and Sysmex Shanghai Medical Electronics Co., Ltd. (Shanghai, China), respectively. The ME-204 analytical balance was from Mettler-Toledo Instrument Co., Ltd. (Shanghai, China). Microscopic observations were performed using an E200 optical microscope (Nikon, Tokyo, Japan). Additional equipment included a centrifuge (Eppendorf, Germany), a DR200B microplate reader (Wuxi Hiwell-Diatech Instruments Co., Ltd., Wuxi, China), an HF-48 high-throughput tissue homogenizer (Shanghai Hefan Instrument Co., Ltd., Shanghai, China), and a JY96-IIN ultrasonic cell disruptor (Shanghai Huxi Industrial Co., Ltd., Shanghai, China).

### 2.2. Experimental Animals

#### 2.2.1. Normobaric Closed Hypoxia, Acute Hypoxia, and Sodium Nitrite Hypoxia Models in Mice

Normobaric Closed Hypoxia Model: Fifty male KM mice (18–20 g) were acclimatized for 3 days and then randomly divided into five groups (n = 10 per group): blank control (Ctrol), acetazolamide (ACT, 100 mg/kg), low-dose trans-sodium crocetinate (TSC-L, 15 mg/kg), medium-dose TSC (TSC-M, 30 mg/kg), and high-dose TSC (TSC-H, 60 mg/kg). All groups received intraperitoneal injections at a volume of 10 mL/kg. Mice were housed under controlled conditions (temperature: 23 °C, relative humidity: 60%) with a 12 h light/dark cycle and free access to sterile food and water. After 5 days of continuous administration under normoxic conditions, mice were placed in 250 mL glass jars containing 5 g soda lime (to absorb CO_2_ and moisture) immediately after the final dose. The jar was tightly sealed, and survival time was recorded from sealing until respiratory arrest. The survival time extension rate (%) was calculated as follows: Survival time extension rate (%) = [Survival time (treatment group) − Survival time (Ctrol)]/Survival time (Ctrol) × 100%.

#### 2.2.2. High-Altitude Acute Lung Injury (HALI) Rat Model

Forty-eight male SD rats (180–220 g) were acclimatized for 3 days and randomly divided into six groups (n = 8 per group): Ctrol, HALI model (Model), ACT (70 mg/kg), TSC-L (10 mg/kg), TSC-M (20 mg/kg), and TSC-H (40 mg/kg). All groups received intraperitoneal injections (2.5 mL/kg). Ctrol and Model groups received saline. After 4 days of normoxic conditioning and prophylactic dosing, non-Ctrol groups were placed in a hypobaric hypoxia chamber simulating rapid ascent to 7000 m (10 m/s) and maintained for 3 days. During hypoxia, dosing continued via a buffer chamber (ascending to 3500 m at 5 m/s for researcher access). On day 8, rats were anesthetized with pentobarbital (30 mg/kg), and blood and lung tissues were collected at 3500 m for analysis.

### 2.3. Hematological Analysis

Blood samples (1 mL) were drawn from the abdominal aorta into heparinized tubes and gently inverted to ensure homogeneity. Hematological analysis was performed using an automated hematology analyzer to determine white blood cell count (WBC), red blood cell count (RBC), hemoglobin (HGB), hematocrit (HCT), platelets (PLT), lymphocytes (LYMPH), and neutrophils (NEUT).

### 2.4. Blood Gas Analysis

Arterial blood (0.6 mL) was drawn from the abdominal aorta via heparinized syringe, promptly sealed with a rubber stopper, and analyzed within 5 min using a DGDR-10II dry electrochemical test strip and a BGA-102 portable blood gas analyzer. Measured parameters included pH, PCO_2_, lactate, PO_2_, [H^+^], actual and standard bicarbonate, base excess (actual and standard), buffer base, SO_2_, and PO_2_/FIO_2_ ratio.

### 2.5. Histopathological Analysis of Lung Tissue via Hematoxylin and Eosin (H&E) Staining

After harvesting the lung tissues, we uniformly infused 4% paraformaldehyde into the lungs through tracheal intubation at a constant pressure of 0.2 MPa. The infusion was performed slowly until the lung tissues were evenly inflated to a physiological filling state, after which they were immersed in the same fixative for subsequent fixation. Lung tissue specimens embedded in paraffin were sequentially processed through heating, xylene-based paraffin removal, and graded alcohol rehydration prior to hematoxylin/eosin (H&E) staining. Following coverslipping with neutral balsam, microscopic evaluation of pulmonary histopathology was conducted at 200× magnification using brightfield microscopy. A semi-quantitative scoring system was implemented to evaluate three key pathological features: (1) peribronchial and parenchymal leukocyte accumulation, (2) thickening of interalveolar septa, and (3) excessive mucus production in airways.

(1) Inflammatory infiltration: ① no infiltration: 0 points; ② mild infiltration (occasional inflammatory cell foci): 1 point; ③ moderate infiltration (1–3 layers of inflammatory cells in most alveoli, bronchi, or vessel walls): 2 points; ④ severe infiltration (>3 layers of inflammatory cells in most alveoli, bronchi, or vessel walls): 3 points [11].

(2) Alveolar septal thickening: ① single-cell layer, no thickening, intact alveolar structure: 0 points; ② mild thickening (1–3× normal thickness, occasional alveolar wall rupture): 1 point; ③ moderate thickening (3–5× normal thickness, alveolar wall rupture): 2 points; ④ severe thickening (>5× normal thickness, extensive alveolar wall damage): 3 points [11].

(3) Airway secretions: ① no secretions: 0 points; ② sparse secretions: 1 point; ③ patchy secretions: 2 points; ④ band-like secretions: 3 points [12].

The average score from five randomly selected fields per slide was calculated as the final result.

### 2.6. Measurement of Inflammatory and Oxidative Stress Markers in Lung Tissue

Lung tissues from standardized locations were rinsed with pre-cooled saline to remove residual blood, blotted dry, and stored at −80 °C. Tissues were weighed, homogenized in 9 volumes of saline to prepare 10% homogenates, and centrifuged. Protein concentrations in the supernatants were determined using a BCA assay kit. Contents of TNF-α, MCP-1, IL-10, MDA, and GSH, as well as SOD activity, were measured strictly according to the instructions of the respective ELISA kits.

### 2.7. Cell Viability Assay

Human pulmonary microvascular endothelial cells (HPMECs) were cultured in DMEM supplemented with 10% fetal bovine serum (FBS) at 37 °C under 5% CO_2_. Cells in the logarithmic growth phase were harvested as single-cell suspensions and seeded into 96-well plates at 5000 cells/well for 24 h. To determine the half-lethal concentration (LD_50_) of CoCl_2_, cells were treated with CoCl_2_ (0, 50, 100, 200, 400, or 800 μmol/L, dissolved in DMSO with 2% FBS) for 12 h (n = 6 replicates per group), followed by CCK-8 assay. Subsequently, HPMECs were exposed to CoCl_2_ at LD_50_ in 2% low-serum medium for 6, 12, or 24 h to identify the optimal modeling duration.

To assess TSC’s cytotoxicity, HPMECs were treated with TSC (6.25, 12.5, 25, 50, 100, or 200 μmol/L) for 12 h. To evaluate TSC’s protective effects against CoCl_2_-induced injury, cells were co-treated with TSC (6.25–200 μmol/L) and 800 μmol/L CoCl_2_ in 2% low-serum medium for 12 h. Cell viability was measured using the CCK-8 kit.

### 2.8. Calcein Acetoxymethylester/Propidium Iodide (Calcein-AM/PI) Staining Assay

HPMECs were seeded into 6-well plates and cultured in a 37 °C, 5% CO_2_ incubator. When cell density reached 70–80%, the old medium was replaced with 2% low-serum medium containing 800 μmol/L CoCl_2_ and different concentrations of TSC (12.5, 25, or 50 μmol/L) for 12 h. After treatment, cells were incubated with a Calcein-AM/PI working solution (prepared according to the kit instructions) at 37 °C in the dark for 30 min. Calcein-AM stains live cells with green fluorescence, while PI labels dead cells with red fluorescence. Images were captured using a fluorescence microplate reader.

### 2.9. Measurement of Lactate (LACT) and Lactate Dehydrogenase (LDH) Release

HPMECs were seeded into 6-well plates and subjected to treatment. After intervention, cell culture medium was collected into sterile centrifuge tubes and centrifuged at 3500 rpm for 15 min. The supernatant was aspirated, and LDH and LACT contents were measured using an automated biochemistry analyzer.

### 2.10. Detection of Cellular Reactive Oxygen Species (ROS) Contents

Human pulmonary microvascular endothelial cells were cultured in confocal imaging dishes until reaching 70–80% density. Following experimental treatments, the culture medium was removed and cells were rinsed twice with phosphate-buffered saline (PBS). The fluorescent probe DCFH-DA was prepared at a 1:1000 dilution in serum-free medium, with 1 mL applied to each culture dish. After 20 min incubation at 37 °C in darkness, cells were PBS-washed and immediately subjected to fluorescence imaging. Quantitative analysis of intracellular ROS contents was performed by measuring fluorescence intensity using ImageJ 1.8.0 processing software.

### 2.11. Measurement of Mitochondrial Membrane Potential

HPMECs in the logarithmetic growth phase were seeded into confocal dishes. After drug intervention, the medium was removed, and cells were washed with JC-1 staining buffer. Fresh medium and JC-1 working solution were added, mixed gently, and incubated for 15 min. After washing with JC-1 buffer, cells were observed and photographed under a confocal microscope.

### 2.12. Detection of Inflammatory Cytokines and Oxidative Stress Markers in Cells

HPMECs were seeded into 6-well plates and cultured in a 37 °C, 5% CO_2_ incubator. At 70–80% confluence, the old medium was replaced with 2% low-serum medium containing TSC (12.5, 25, or 50 μmol/L) for 12 h. Cell supernatants were collected, and contents of TNF-α, MCP-1, IL-10, MDA, GSH, and SOD activity were measured using ELISA kits according to the manufacturer’s instructions.

### 2.13. Network Pharmacology Study

#### 2.13.1. Target Acquisition for TSC and HALI

The two-dimensional molecular structure of trans-sodium crocetinate (TSC) was acquired from PubChem and subsequently submitted to PharmMapper for potential target identification. Following target prediction, protein standardization was performed using UniProt to eliminate non-relevant targets. Concurrently, disease-associated targets for high-altitude acute lung injury (HALI) were systematically collected from GeneCards and OMIM databases through keyword searching. After removal of redundant entries, the overlapping targets between TSC and HALI were determined via Venny 2.1 (https://bioinfogp.cnb.csic.es/tools/venny/, accessed on 15 April 2025), establishing a putative target profile for TSC’s therapeutic effects against HALI.

#### 2.13.2. Protein/Protein Interaction (PPI) Network Construction and Core Target Screening

The putative therapeutic targets of TSC for HALI intervention were analyzed using the STRING platform (version 11.5; https://string-db.org/, accessed on 15 April 2025) to establish a protein interaction network, with the interaction threshold set to ≥0.4 confidence score. The network data were exported in TSV format and subsequently imported into Cytoscape (v3.7.2) for network visualization and further analysis. Network topology parameters were calculated using the CytoNCA plugin, with key hub targets identified through a systematic screening approach where nodes demonstrating degree centrality values surpassing twice the median network value were selected as core targets.

#### 2.13.3. Gene Ontology (GO) and Kyoto Encyclopedia of Genes and Genomes (KEGG) Pathway Enrichment Analysis

Functional annotation of potential targets was conducted using the DAVID database (https://david.ncifcrf.gov, accessed on 16 April 2025) for GO terms and KEGG pathways, ranked by q-value. Results were visualized using the Bioinformatics Platform (https://www.bioinformatics.com.cn/, accessed on 16 April 2025).

### 2.14. Molecular Docking

Five key targets (EGFR, IGF-1, PIK3CA, AKT1, and NF-κB1) were prioritized for molecular docking with trans-sodium crocetinate (TSC) through integrated KEGG pathway analysis and comprehensive literature evaluation. The ligand structure (TSC) was retrieved from PubChem in SDF format, while three-dimensional protein structures were acquired from RCSB PDB (PDB format). Protein preparation involved removal of crystallographic water molecules and co-crystallized ligands using PyMOL molecular visualization system. Structure conversion to PDBQT format and binding site identification were performed using AutoDock Tools (version 1.5.6). The docking simulations were executed with AutoDock Vina (v1.1.2), employing a Lamarckian genetic algorithm, with subsequent visualization and analysis conducted in Discovery Studio 2019 and PyMOL (v2.5).

### 2.15. Molecular Dynamics (MD) Simulation Validation

The molecular dynamics simulations were carried out for 100 nanoseconds using GROMACS 2022 software package. The protein/ligand complexes were prepared with CHARMM36 all-atom force field for biomolecules and General Amber Force Field 2 (GAFF2) for the small molecule. The system was immersed in a cubic water box (1.2 nm periodic boundary) employing the TIP3P explicit solvent model. Long-range electrostatic interactions were treated with Particle Mesh Ewald (PME) summation, while the Verlet cut-off scheme was implemented for integration. System equilibration involved two phases: (1) 100 ps NVT ensemble with velocity rescaling thermostat (τ = 0.1 ps), followed by (2) 100 ps NPT ensemble using Parrinello–Rahman barostat (τ = 0.1 ps). Non-bonded interactions were calculated with a 1.0 nm cut-off for both van der Waals and Coulombic terms. Production simulations were performed under physiological conditions (310 K, 1 bar) for 100 ns duration.

### 2.16. ELISA Validation of EGFR, PI3K, AKT, and NF-κB Expression in HALI Rat Lung Tissue

Lung tissues from standardized locations were rinsed with pre-cooled saline, blotted dry, and stored at −80 °C. Tissues were homogenized in 9 volumes of saline to prepare 10% homogenates. Protein concentrations were measured using a BCA assay kit. Contents of EGFR, PI3K, AKT, and NF-κB were quantified via ELISA according to the manufacturer’s protocols.

### 2.17. RT-qPCR Validation of EGFR, PI3K, AKT, and NF-κB mRNA Expression in HALI Rat Lung Tissue

Total RNA was extracted from lung tissues using a total RNA extraction kit, reverse-transcribed into cDNA, and amplified by PCR. GAPDH served as the internal reference gene. mRNA expression contents of EGFR, PI3K, AKT, and NF-κB were calculated using the 2^−ΔΔCt^ method. All primers were designed and synthesized by Beijing Liuhe BGI Gene Technology Co., Ltd. (Beijing, China; primer sequences listed in Table 1).

### 2.18. Statistical Analysis

Data are expressed as mean ± standard deviation (SD). Normality of the data was rigorously assessed prior to statistical analysis: quantitatively via the Shapiro–Wilk test (with *p* > 0.05 indicating a normal distribution) and visually through Q-Q plots and histograms. For normally distributed data, differences between multiple groups were analyzed using one-way ANOVA, followed by Tukey’s post hoc test. A *p* < 0.05 was considered statistically significant. Analyses and graphs were generated using GraphPad Prism 8.3.0.

## 3. Results

### 3.1. Effects of TSC on Hypoxia-Induced Survival Time in Mice

**Figure 1:**
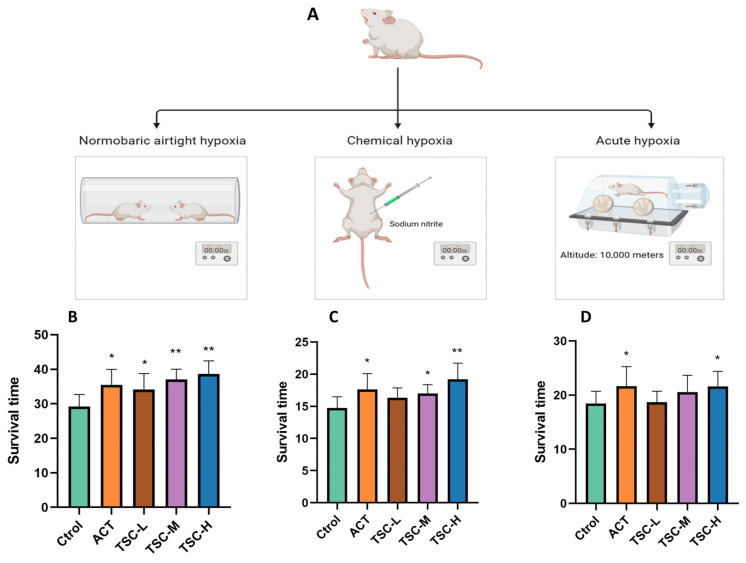
Effects of TSC on hypoxia-induced survival time in mice. Data are expressed as mean ± SD (n = 10 per group). Note: Compared with the Ctrol group, * *p* < 0.05, ** *p* < 0.01. Ctrol: Control group; ACT: Positive control group (acetazolamide); TSC-L: Low-dose TSC group; TSC-M: Medium-dose TSC group; TSC-H: High-dose TSC group (the same applies below). (**A**) Schematic diagram of the hypoxia model experimental protocol; (**B**) normobaric closed hypoxia test; (**C**) sodium nitrite-induced hypoxia test; (**D**) acute hypoxia test.

In the normobaric closed hypoxia model, compared with the Ctrol group, the ACT group and all TSC dose groups significantly prolonged the survival time of mice under hypoxia (*p* < 0.05), with survival time extension rates of 21.47%, 16.87%, 26.75%, and 32.30%, respectively (Figure 1B).

In the chemical hypoxia model (sodium nitrite-induced), the ACT group and the medium- and high-dose TSC groups markedly extended survival time (*p* < 0.05), with extension rates of 20.41%, 14.93%, and 27.49%, respectively (Figure 1C).

In the acute hypoxia model (simulated altitude ascent), the ACT group and the high-dose TSC group significantly prolonged survival time (*p* < 0.05), achieving extension rates of 17.41% and 17.04%, respectively (Figure 1D). These results demonstrate that TSC exhibits significant anti-hypoxic activity.

### 3.2. Effects of TSC on Hematological Parameters in HALI Rats

**Figure 2:**
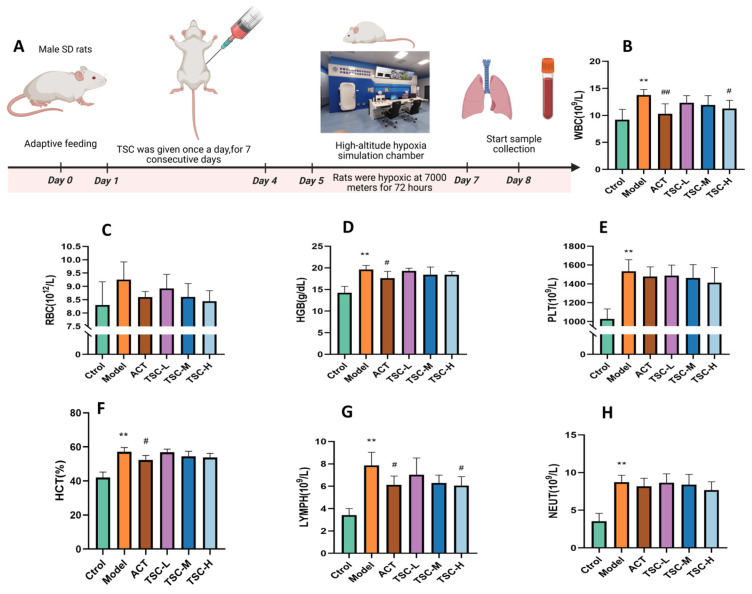
Effects of TSC on hematological parameters in HALI rats. Data are expressed as mean ± SD (n = 8 per group). Notes: Compared with the Ctrol group: ** *p* < 0.01. Compared with the Model group: # *p* < 0.05, ## *p* < 0.01, Ctrol: Control group; ACT: Positive control group (acetazolamide); TSC-L: Low-dose TSC group; TSC-M: Medium-dose TSC group; TSC-H: High-dose TSC group. (**A**) Schematic diagram of the HALI rat model experimental protocol. (**B**) WBC: White blood cell concentration; (**C**) RBC: Red blood cell concentration; (**D**) HGB: Hemoglobin concentration; (**E**) PLT: Platelet concentration; (**F**) HCT: Hematocrit ratio; (**G**) LYMPH: Lymphocyte concentration; (**H**) NEUT: Neutrophil concentration.

Results showed that compared with the Ctrol group, the Model group exhibited significant increases in WBC, RBC, HGB, PLT, LYMPH, NEUT (*p* < 0.05 or *p* < 0.01), and hematocrit (HCT, *p* < 0.01). Compared with the Model group, all TSC dose groups reduced the concentrations of WBC, RBC, HGB, HCT, PLT, LYMPH, and NEUT in HALI rats to varying degrees (Figure 2B–H). Notably, the high-dose TSC group (TSC-H) showed the most significant suppression of WBC and LYMPH (*p* < 0.05).

### 3.3. Effects of TSC on Blood Gas Indicators in HALI Rats

**Figure 3:**
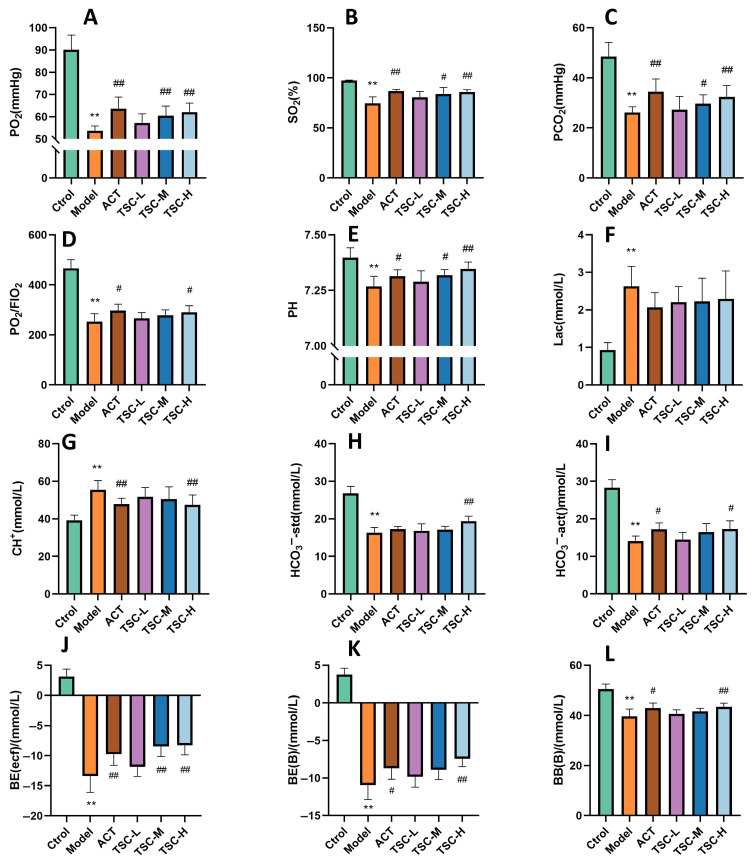
The effect of TSC on blood gas indicators in HALI rats. The data is expressed as Mean ± SD (n = 8/group). Note: Compared with the Ctrol group, ** *p* < 0.01. Compared with the Model group, # *p* < 0.05, ## *p* < 0.01. (**A**) PO_2_: Arterial oxygen partial pressure; (**B**) SO_2_: Blood oxygen saturation; (**C**) PCO_2_: partial pressure of carbon dioxide in arterial blood; (**D**) PO_2_/FIO_2_: Arterial oxygen partial pressure/fraction of oxygen concentration in inhaled gas; (**E**) PH: acidity and alkalinity; (**F**) Lac: Lactic acid; (**G**) CH^+^: hydrogen ion concentration; (**H**) HCO_3_^−^-std: standard bicarbonate; (**I**) HCO_3_^−^-act: actual bicarbonate; (**J**) BE: Remaining standard alkali; (**K**) BE (B): Actual alkali residue; (**L**) BB (B): Buffer base.

The results showed that compared with the Ctrol group, the contents of PO_2_, SO_2_, PCO_2_, PO_2_/FIO_2_, PH, HCO_3_^−^-std, HCO_3_^−^-act, BE (ecf), BE (B), and BB (B) in HALI rats decreased (*p* < 0.01), while the contents of Lac and CH^+^ increased (*p* < 0.01), indicating that the high-altitude low-pressure hypoxic environment can lead to a decrease in blood oxygen partial pressure and acid/base balance disorder in HALI rats. Compared with the Model group, each dose group of TSC can reverse the contents of the above indicators to varying degrees (*p* < 0.05 or *p* < 0.01) (Figure 3A–L), indicating that TSC can increase blood oxygen partial pressure and maintain acid/base balance in HALI rats.

### 3.4. Effects of TSC on the Pathology of Lung Tissue in HALI Rats

**Figure 4:**
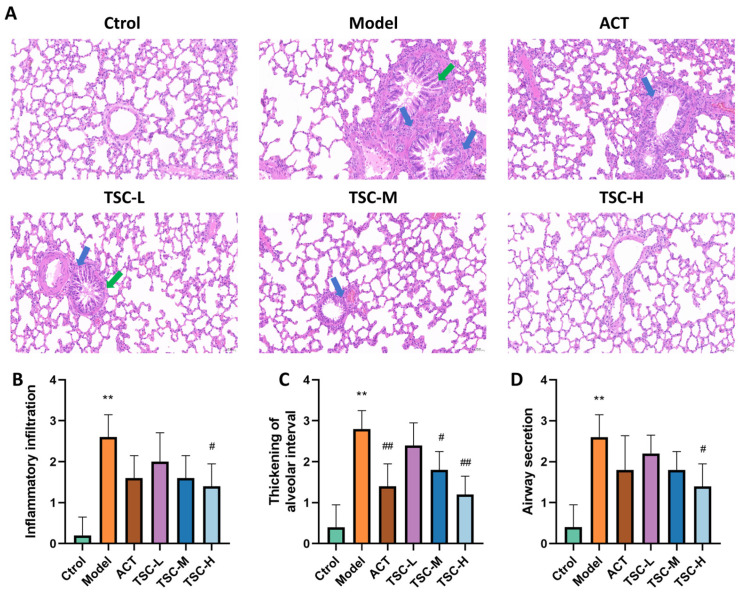
Effects of TSC on lung tissue pathology in HALI rats. Note: Compared with the Ctrol group, ** *p* < 0.01; compared with the Model group, # *p* < 0.05, ## *p* < 0.01. Image magnification: 200×, scale: 50 μm. The blue arrow represents infiltration of inflammatory cells, and the green arrow represents pulmonary tissue edema. (**A**) HE pathological diagram; (**B**) Lung tissue inflammatory infiltration score; (**C**) Thickening of alveolar interval score; (**D**) Airway secretion score.

The pathological results of lung tissue showed that the alveolar structure of the Ctrol group rats was intact, and no significant pathological changes were observed in the airway and surrounding lung tissue (Figure 4A). Compared with the Ctrol group, the lung tissue structure of the Model group rats was disordered, with increased alveolar septum thickness, alveolar wall rupture and fusion into pulmonary bullae, narrow lumen, epithelial cell shedding and increased mucus secretion in the lumen, and a large number of inflammatory cell infiltration in various layers of the mucosa. The pathological score of lung tissue was significantly increased (*p* < 0.01) (Figure 4A–D). Compared with the Model group, the pathological changes in lung tissue were improved to varying degrees in the TSC dose groups, and the pathological scores of lung tissue were significantly reduced (*p* < 0.05 or *p* < 0.01) (Figure 4A–D).

### 3.5. EeffectS of TSC on Inflammation and Oxidative Stress Indicators in Lung Tissue of HALI Rats

**Figure 5:**
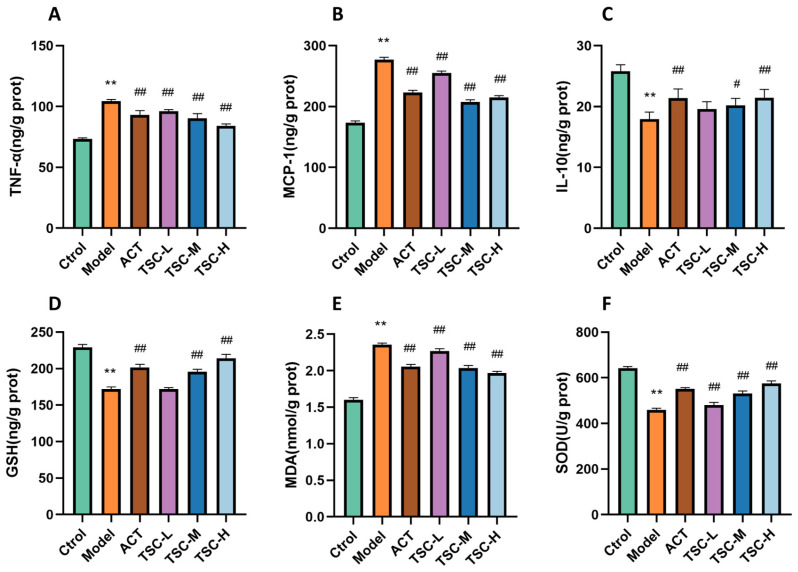
Effects of TSC on inflammation and oxidative stress indicators in lung tissue of HALI rats. Note: Compared with the Ctrol group, ** *p* < 0.01; compared with the Model group, # *p* < 0.05, ## *p* < 0.01. (**A**) TNF-α: tumor necrosis factor-α; (**B**) MCP-1: monocyte chemoattractant protein-1; (**C**) IL-10: interleukin-10; (**D**) GSH: glutathione; (**E**) MDA: malondialdehyde; (**F**) SOD: Superoxide Dismutase.

Compared with the Ctrol group, the contents of TNF-α, MCP-1, and MDA in the lung tissue of the Model group rats were significantly increased (*p* < 0.01), while the contents of IL-10 and GSH were significantly decreased (*p* < 0.01), and the activity of SOD was significantly decreased (*p* < 0.01) (Figure 5). Compared with the Model group, the TSC dose groups were able to reduce TNF-α, MCP-1, and MDA contents (*p* < 0.01), increase IL-10 and GSH contents (*p* < 0.01), and increase SOD activity (*p* < 0.01) (Figure 5), indicating that TSC can alleviate HALI injury by inhibiting inflammatory response and oxidative stress.

### 3.6. Effects of TSC on COCl_2_-Induced Hypoxia Damage in HPMEC

**Figure 6:**
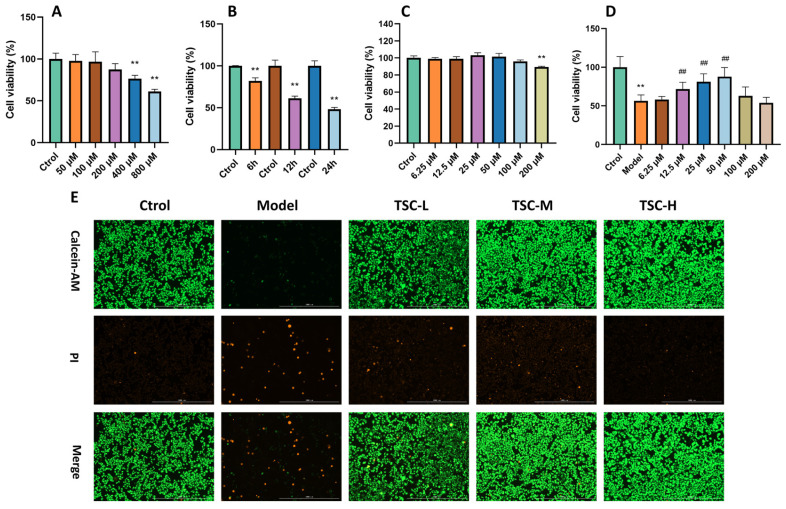
Effect of TSC on COCl_2_-induced hypoxia damage in HPMECs. Note: Compared with the Ctrol group, ** *p* < 0.01; compared with the Model group, ## *p* < 0.01. (**A**–**D**) Cell ability; (**E**) Calcein AM/PI staining, image magnification: 4×, scale: 1000 μm.

Treating HPMECs with different concentrations of CoCl_2_ for 12 h resulted in a concentration-dependent decrease in cell viability (Figure 6A), with 800 mol/L CoCl_2_ reducing cell viability to around 60% (*p* < 0.01). Therefore, this concentration was chosen for subsequent modeling of cellular hypoxia injury. Using 800 μmol/L CoCl_2_ to intervene in cells for 6 h, 12 h, and 24 h, it was found that after 12 h of treatment, cell viability decreased to 60% (*p* < 0.01) (Figure 6B), which was moderate and controllable. Therefore, in subsequent experiments, 800 μmol/L CoCl_2_ was used to treat for 12 h to establish an HPMEC hypoxia injury model.

Preliminary verification of the toxicity of TSC to HPMECs showed that after treating HPMECs with TSC solutions at concentrations of 6.25, 12.5, 25, 50, 100, and 200 μmol/L for 12 h, the cell viability remained above 80% (Figure 6C), indicating that TSC has a relatively small toxic effect on HPMECs. After treating HPMECs with TSC at the above concentrations and 800 μ mol/L CoCl_2_ for 12 h, TSC at 12.5, 25, and 50 μM significantly improved CoCl_2_-induced hypoxia damage in HPMECs (*p* < 0.01) (Figure 6D). Therefore, concentrations of 12.5, 25, and 50 μM were selected as low (TSC-L), medium (TSC-M), and high doses (TSC-H) of TSC for subsequent experiments. In addition, Calcein AM/PI staining of cells further indicated that TSC could significantly improve CoCl_2_-induced hypoxia damage in HPMECs (Figure 6E).

### 3.7. Effects of TSC on the Contents of Hypoxic LDH and LACT in HPMECs

**Figure 7:**
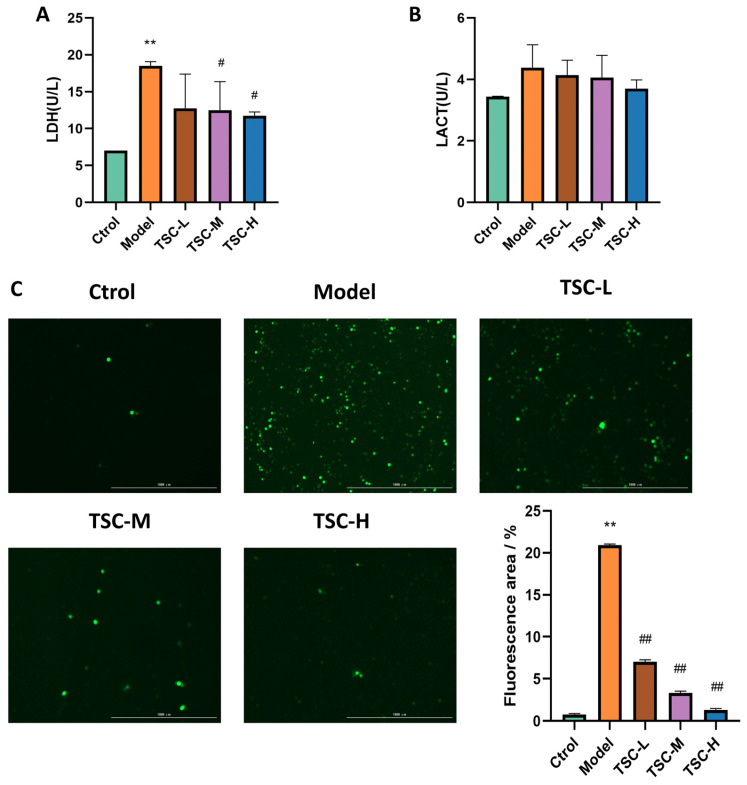
Effects of TSC on hypoxia-induced LDH, LACT, and ROS in HPMECs (4×). Note: Compared with the Ctrol group, ** *p* < 0.01. Compared with the Model group, # *p* < 0.05, ## *p* < 0.01. (**A**) LDH: lactate dehydrogenase; (**B**) LACT: lactic acid; (**C**) ROS fluorescence staining and quantitative analysis. Image magnification: 4×, scale: 1000 μm.

Compared with the Ctrol group, the contents of HPMEC LDH (Figure 7A) and LACT in the Model group were significantly increased (Figure 7B) (*p* < 0.01). Compared with the Model group, each dose group of TSC can reduce LDH (Figure 7A) and LACT contents to varying degrees (Figure 7B). There was a significant difference (*p* < 0.05) between the TSC medium- and high-dose groups.

### 3.8. Effects of TSC on Hypoxia-Induced ROS in HPMECs

Quantitative analysis revealed a marked elevation in ROS fluorescence intensity in HPMECs from the Model group compared to Ctrol (*p* < 0.01, Figure 7C). Treatment with TSC at all tested concentrations demonstrated significant attenuation of hypoxia-induced ROS accumulation relative to the Model group (*p* < 0.01, Figure 7C). These results suggest that TSC exerts protective effects against hypoxic damage through ROS suppression.

### 3.9. Effects of TSC on the Level of Hypoxic Mitochondrial Membrane Potential in HPMECs

**Figure 8:**
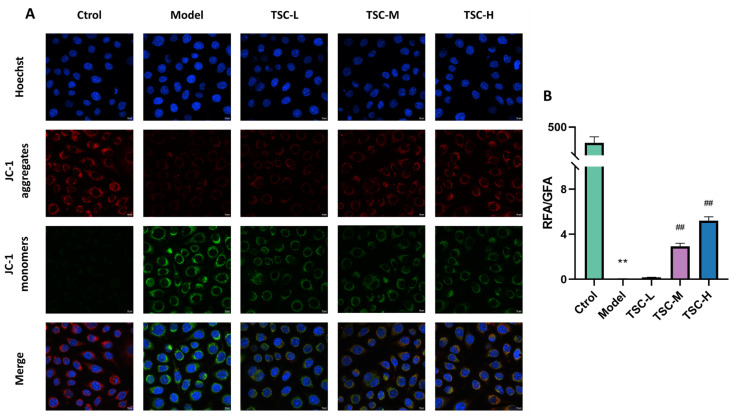
Effect of TSC on the level of mitochondrial membrane potential in HPMECs under hypoxia (100×). Note: Compared with the Ctrol group, ** *p* < 0.01; compared with the Model group, ## *p* < 0.01. RFA: Red fluorescence area; GFA: Green fluorescence area. Image magnification: 100×, scale: 10 μm. (**A**) Fluorescence staining of mitochondrial membrane potential; (**B**) Quantitative analysis of mitochondrial membrane potential.

Compared with the Ctrol group, the Model group showed a significant decrease in mitochondrial membrane potential (*p* < 0.01). Compared with the Model group, the TSC treatment group (especially TSC-H) significantly restored mitochondrial membrane potential in a dose-dependent manner (*p* < 0.01) (Figure 8A,B). This result suggests that TSC may alleviate hypoxia-induced cell damage by maintaining mitochondrial membrane potential stability.

### 3.10. Effects of TSC on Inflammation and Oxidative Stress Indicators in HPMEC Supernatant

**Figure 9:**
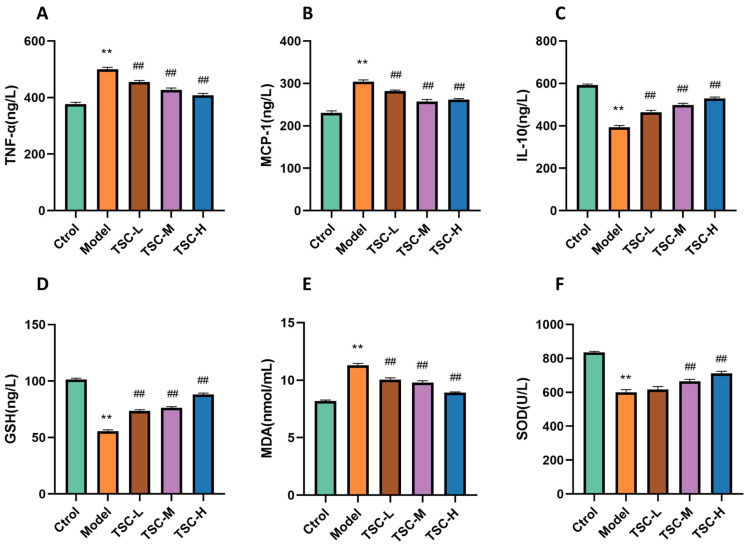
The effect of TSC on inflammation and oxidative stress indicators in HPMEC supernatant. Note: Compared with the Ctrol group, ** *p* < 0.01; compared with the Model group, ## *p* < 0.01. (**A**) TNF-α: tumor necrosis factor-α; (**B**) MCP-1: monocyte chemoattractant protein-1; (**C**) IL-10: interleukin-10; (**D**) GSH: glutathione; (**E**) MDA: malondialdehyde; (**F**) SOD: Superoxide Dismutase.

Compared with the Ctrol group, the Model group showed significantly increased contents of TNF-α, MCP-1, and MDA; significantly decreased contents of IL-10 and GSH (*p* < 0.01); and significantly decreased SOD activity (*p* < 0.01) (Figure 9). Compared with the Model group, each dose group of TSC can reduce the contents of TNF-α, MCP-1, and MDA (*p* < 0.01); increase the contents of IL-10 and GSH (*p* < 0.01); and increase SOD activity (*p* < 0.01) (Figure 9), further confirming that TSC can alleviate cellular hypoxia damage by inhibiting inflammatory response and oxidative stress.

### 3.11. Network Pharmacology Results

**Figure 10:**
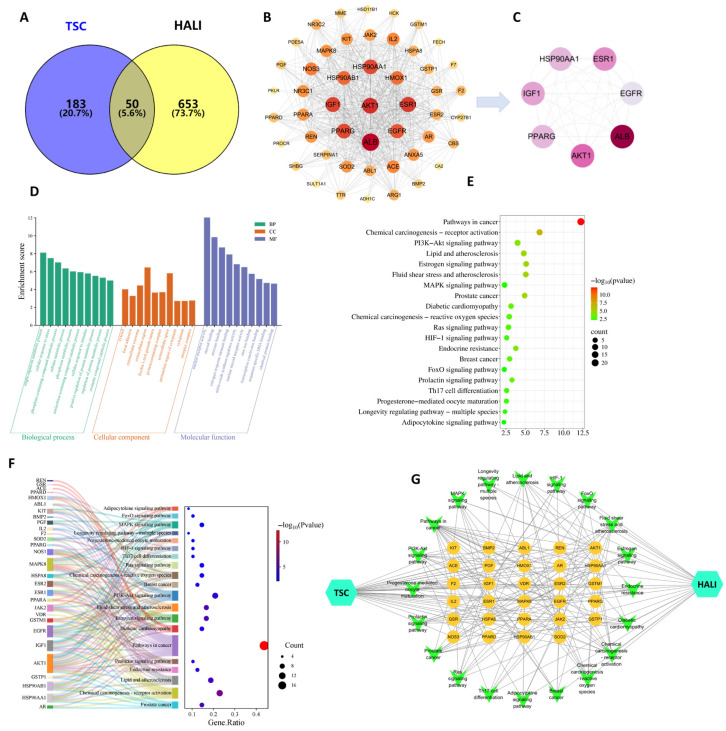
Network pharmacology analysis of TSC treatment for HALI. (**A**) Intersection target Venny plot; (**B**) PPI network diagram; (**C**) core target network diagram; (**D**) GO enrichment analysis; (**E**) KEGG enrichment analysis; (**F**) target pathway Sankey bubble plot; (**G**) drug target pathway disease network diagram.

The results of network pharmacology showed that 233 TSC targets, 703 HALI disease targets, and 50 potential intersecting targets for TSC treatment of HALI were obtained through target fishing (Figure 10A). According to topological analysis, the PPI network contains a total of 49 nodes and 710 edges. The darker and larger the color of the nodes in the figure, the higher the correlation (Figure 10B). Selecting the median value greater than 2 degrees (≥26) as the core target, seven core target genes, including EGFR, AKT1, IGF1, PPARG, HSP90AA1, PPARG, and ESR1 were ultimately obtained (Figure 10C).

Enrichment analysis was conducted on 50 potential target genes for TSC treatment of HALI, with gene annotation and classification based on biological processes (BPs), cellular components (CCs), and molecular functions (MFs). A total of 326 GO entries were obtained (*p*-value < 0.05), including 222 BP, 38 CC, and 66 MF entries. Analysis found that BPs are often involved in hypoxia response, signal transduction, and apoptosis regulation response; CCs often involve cytoplasm, extracellular vesicles, etc.; MFs often involve nuclear receptor activity, steroid binding, enzyme binding, etc. (Figure 10D). KEGG pathway analysis of putative target genes identified 66 significantly enriched pathways (*p* < 0.05), with predominant involvement of the PI3K-AKT and MAPK signaling cascades (Figure 10E). In the enrichment visualization, graphical elements (bar height/bubble diameter) represent gene counts, while color intensity corresponds to statistical significance (redder hues indicate lower *p*-values). Subsequent network pharmacology analysis was performed by integrating these targets and pathways with Cytoscape 3.7.2 to generate a comprehensive “compound/target/pathway/disease” interaction network (Figure 10G). The network topology revealed that core targets of TSC modulate multiple signaling pathways, demonstrating its polypharmacological mechanism against high-altitude pulmonary injury.

### 3.12. Molecular Docking Validation

**Figure 11:**
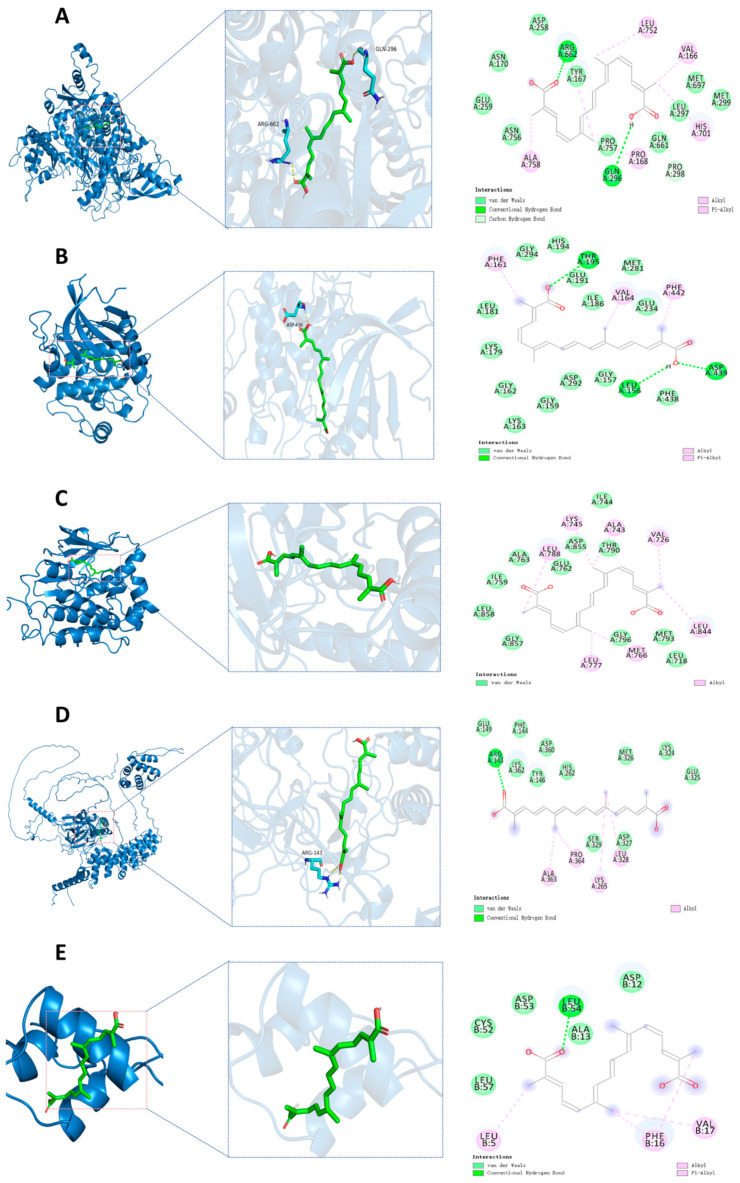
Molecular docking. (**A**) Molecular docking of PIK3CA-TSC; (**B**) molecular docking of AKT1-TSC; (**C**) molecular docking of EGFR-TSC; (**D**) molecular docking of NF-κB1-TSC; (**E**) molecular docking of IGF1-TSC.

By reviewing the relevant literature, PIK3CA, AKT1, EGFR, IGF1, and NF-κB1 were selected as candidate docking targets for molecular docking with TSC. The molecular docking analysis revealed strong binding affinities between the target proteins and active compounds, with all calculated binding energies <−5.0 kcal/mol (Table 2). These robust intermolecular interactions (−5.0 to −9.5 kcal/mol) demonstrate favorable binding activity between the key phytochemicals and core targets, thereby validating the predictive reliability of our network pharmacology approach. The ARG662 and GLN296 residues on the PIK3CA receptor form hydrogen bonds with trTSC; ASP258, ASN170, GLU259, ASN756, PRO757, GLN661, LEU297, MET697, and MET299 residues form van der Waals with TSC; PRO298 residue forms a carbon/hydrogen bond with TSC; ALA758, PRO168, HIS701, VAL166, and LEU752 residues form hydrophobic interactions with trans-sodium crocetinate (Figure 11A). The residues LEU156, ASP439, and THR195 on the AKT1 receptor form hydrogen bonds with TSC; LEU181, LYS179, GLY162, LYS163, GLY159, ASP292, GLY157, PHE438, GLU234, ILE186, MET281, GLU191, HIS194 form van der Waals with GLY294 residue and TSC; PHE161, VAL164, and PHE442 residues form hydrophobic interactions with TSC (Figure 11B). On the EGFR receptor, ILE744, THR790, ASP855, GLU762, ALA763, ILE759, LEU858, GLY857, GLY796, MET793 form van der Waals with LEU718 residue and TSC; LEU788, LYS745, ALA743, VAL726, LEU844, MET766, and LEU777 residues form hydrophobic interactions with TSC (Figure 11C). The ARG143 residue on the NF-κB1 receptor forms hydrogen bonds with TSC. GLU149, LYS362, PHE144, TYR146, ASP360, HIS262, MET326, LYS324, GLU325, ASP327, and SER329 residues form van der Waals with TSC. ALA363, PRO364, LYS265, and LEU328 residues form hydrophobic interactions with TSC (Figure 11D). The LEU54 residue on the IGF-1 receptor forms hydrogen bonds with TSC; LEU57, CYS52, ASP53, ALA13, and ASP12 residues form van der Waals with TSC; LEU5, PHE16, and VAL17 residues form hydrophobic interactions with TSC (Figure 11E).

### 3.13. Molecular Dynamics Simulation Verification

**Figure 12:**
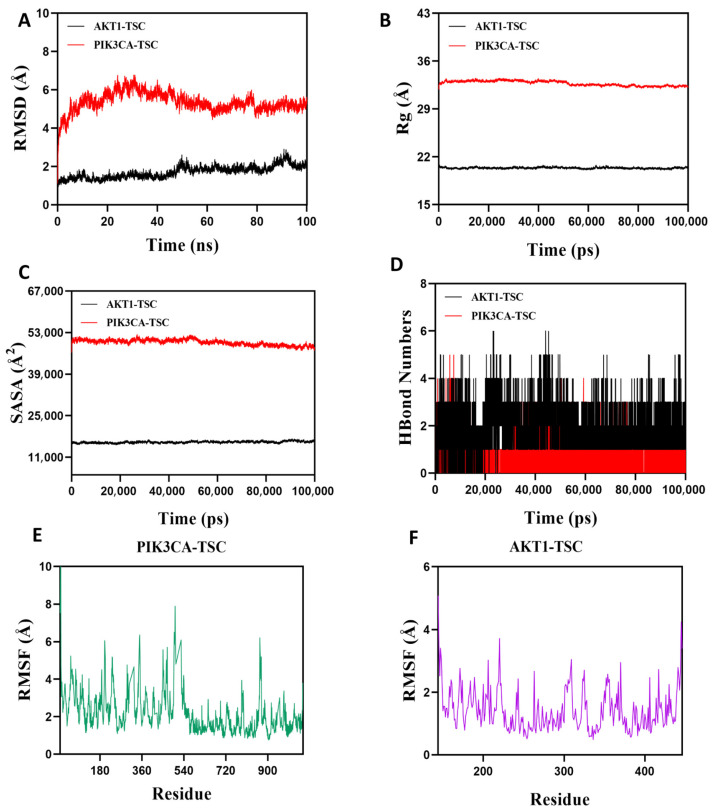
Molecular dynamics simulation verification analysis. (**A**) RMSD values of protein/ligand complexes over time. (**B**) Rg value of protein/ligand complexes over time. (**C**) SASA values of protein/ligand complexes over time. (**D**) HBonds values of protein/ligand complexes over time. RMSF values of amino acid backbone atoms of (**E**,**F**) protein/ligand complexes over time.

Root Mean Square Deviation (RMSD) is an effective metric for assessing the conformational stability of proteins and ligands, as well as the degree of deviation of atomic positions from their initial positions. A smaller deviation indicates better conformational stability, so this study used RMSD to evaluate the equilibrium state of the simulation system. As shown in Figure 12A, the AKT1-TSC complex system exhibited stable fluctuations between 5 ns and 50 ns. Although there was a slight upward trend after 50 ns, the overall fluctuation amplitude remained below 2.2 Å. The PIK3CA-TSC complex system reached equilibrium after 80 ns and finally stabilized with fluctuations around 5.3 Å. It can be seen that TSC has high stability when bound to AKT1 and PIK3CA target proteins. The radius of gyration (Rg) can be used to describe the changes in the overall structure of proteins and characterize the tightness of their structures. A larger change in Rg indicates a looser system. During the movement, the Rg values of both AKT1-TSC and PIK3CA-TSC complexes showed relatively stable fluctuations, indicating that the complexes formed by small molecules and target proteins did not undergo significant expansion or contraction during movement (Figure 12B). The solvent-accessible surface area (SASA) is an important indicator for evaluating the surface area of proteins. In this simulation, the solvent-accessible surface area between the target protein and small molecules was calculated. Figure 12C shows that after the receptor and ligand binding, the SASA of both AKT1-TSC and PIK3CA-TSC complexes did not change significantly, which indicates that ligand binding has little impact on the protein structure. Hydrogen bonds play a key role in the binding process between ligands and proteins. Figure 12D shows the changes in the number of hydrogen bonds between small molecules and target proteins during the dynamic process. In the AKT1-TSC complex system, the number of hydrogen bonds varies from 0 to 6, with the complex maintaining approximately four hydrogen bonds in most instances. For the PIK3CA-TSC complex system, the number of hydrogen bonds ranges from 0 to 4, and it typically has around three hydrogen bonds. These observations indicate favorable hydrogen bond interactions in both systems. Root mean square fluctuation (RMSF) is a parameter that reflects the flexibility of amino acid residues within proteins. The RMSF values of both the AKT1-TSC and PIK3CA-TSC complexes are relatively low, mostly staying below 6 Å. This low RMSF indicates that the amino acid residues in these complexes have low flexibility, which in turn implies high stability (Figure 12E,F).

In summary, the AKT1-TSC and PIK3CA-TSC complex system has stable binding and good hydrogen bonding properties. Therefore, TSC binds well to AKT1 and PIK3CA target proteins.

### 3.14. ELISA Validation of EGFR, PI3K, AKT, and NF-κB Expression Contents

**Figure 13:**
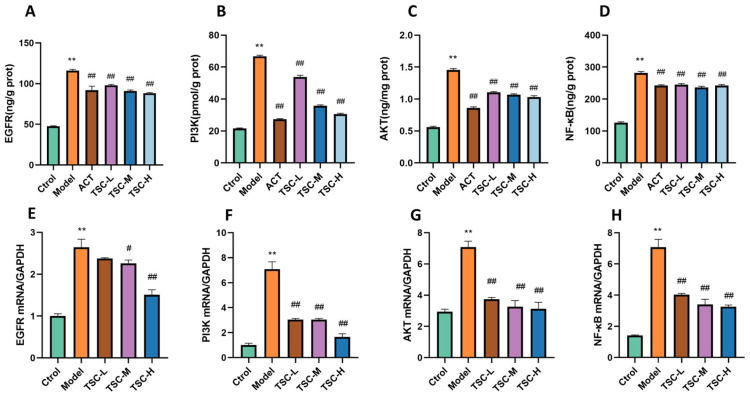
The effect of TSC on the EGFR/PI3K/AKT/NF-κB signaling pathway. Note: Compared with the Ctrol group, ** *p* < 0.01; compared with the Model group, # *p* < 0.05, ## *p* < 0.01. (**A**) EGFR: Epidermal growth factor receptor; (**B**) PI3K: Phosphatidylinositol-3-kinase; (**C**) AKT: Protein kinase B; (**D**) NF-κB: Nuclear transcription factor. (**E**–**H**) Expression levels of EGFR, PI3K, AKT, and NF-κB mRNA.

Compared with the Ctrol group, the contents of EGFR, PI3K, AKT, and NF-κB were significantly increased in the Model group (*p* < 0.01) (Figure 13A–D). Compared with the Model group, the TSC dose groups were able to reduce the contents of EGFR, PI3K, AKT, and NF-κB (*p* < 0.01) (Figure 13A–D).

### 3.15. RT qPCR Validation of EGFR, PI3K, AKT, and NF-κB mRNA Expression

The results showed that compared with the Ctrol group, the mRNA contents of EGFR, PI3K, AKT, and NF-κB were significantly increased in the Model group (*p* < 0.01) (Figure 13E–H). Compared with the Model group, the TSC dose groups were able to reduce the mRNA expression of EGFR, PI3K, AKT, and NF-κB (*p* < 0.01) (Figure 13E–H), further confirming that TSC can alleviate acute lung injury induced by high-altitude hypoxia by regulating the EGFR/PI3K/AKT/NF-κB signaling pathway.

## 4. Discussion

As an inflammatory disease with a difficult cure and poor prognosis, acute lung injury is characterized by high incidence rate and high mortality. The high-altitude low-pressure and hypoxic environment can cause a series of pathological and physiological changes in human tissues and organs due to hypoxia, which is one of the important causes of acute lung injury [13]. Research has shown that rapid exposure to high-altitude hypoxia can directly lead to a decrease in inhaled oxygen partial pressure (PiO_2_), causing a decrease in alveolar oxygen partial pressure, hindering the diffusion of oxygen molecules into the bloodstream, and ultimately resulting in a decrease in arterial oxygen partial pressure (PO_2_) and oxygen saturation (SO_2_) [14]. Low PO_2_ stimulates the respiratory center, triggering the drive for low oxygen ventilation, leading to deepening and accelerating breathing, thereby reducing PCO_2_ and further inducing hypocapnia and respiratory alkalosis [15]. In addition, the total hemoglobin (THb) concentration in the body gradually increases with the prolongation of hypoxia time, but the proportion of oxygenated hemoglobin (O2Hb) shows a decreasing trend, leading to a decrease in oxygen transport efficiency and further exacerbating lung tissue hypoxia damage [16]. Therefore, acute hypoxia at high altitudes can lead to multiple pathological and physiological cascade reactions such as decreased oxygen partial pressure and acid/base imbalance in the body. The results of this study indicate that TSC can significantly improve the respiratory metabolic acid/base imbalance and enhance the hemoglobin oxygen transport capacity in HALI rats. In vitro experiments further showed that TSC can significantly improve CoCl_2_-induced hypoxia damage in HPMECs. The inflammatory response mediated by inflammatory cells and inflammatory active substances is a key link in the occurrence of HALI injury. High-altitude hypoxia stimulation changes the immune microenvironment of the body, inducing a large number of inflammatory cells such as neutrophils and macrophages to adhere to and infiltrate lung tissue, and releasing various inflammatory factors (TNF-α, MCP-1, etc.), neutrophil elastase, and other inflammatory mediators [17,18]. These inflammatory cells interact with inflammatory mediators, forming an “inflammatory waterfall” effect and disrupting the alveolar capillary barrier. Under low-oxygen conditions, there is an imbalance between lung tissue oxidation and antioxidant activity, leading to excessive generation of reactive oxygen species (ROS) and causing lipid peroxidation damage and DNA oxidative damage to the biological membrane, further exacerbating alveolar epithelial cell apoptosis and pulmonary interstitial fibrosis [19]. The above pathological processes are intertwined, ultimately forming acute lung injury characterized by imbalance of lung ventilation blood flow ratio, diffusion dysfunction, and decreased lung compliance. The results of this study found that TSC can significantly reduce the contents of TNF-α, MCP-1, and MDA; increase IL-10 and GSH contents; and improve SOD activity, indicating that TSC can alleviate HALI injury by inhibiting inflammatory response and oxidative stress. Furthermore, IL-1β and the NLRP3 inflammasome are key molecules with high specificity in innate immune activation and downstream effects of the NF-κB pathway. Future studies will explore the possibility that TSC exerts its effects by regulating the NF-κB/NLRP3/IL-1β axis.

The occurrence and development of HALI are closely related to mitochondrial dysfunction, among which the dynamic changes in mitochondrial membrane potential are a key link connecting hypoxia stress and cell damage [20]. Low-oxygen environment interferes with mitochondrial energy metabolism, redox balance, and membrane structure stability through multiple pathways, ultimately leading to abnormal fluctuations in mitochondrial membrane potential, triggering cell apoptosis and inflammatory cascade reactions [21]. Huan et al. [22] induced an acute high-altitude cerebral hypoxia injury model in mice through a low-pressure hypoxia chamber and found that after high-altitude hypoxia, the mitochondrial membrane potential and adenosine triphosphate production of mouse neurons decreased, while oxidative stress and mitochondrial fission increased. Li et al. [23] found in an in vitro model of hypoxic myocardial injury that the mitochondrial membrane potential of cells was significantly reduced, while quercetin could restore the mitochondrial membrane potential level of hypoxic myocardial injury cells. Similarly, in the CoCl_2_-induced HPMEC hypoxia injury model used in this study, it was observed that the mitochondrial membrane potential level was significantly reduced in the Model group, while TSC administration could reverse the mitochondrial membrane potential level. This suggests that TSC may alleviate hypoxia-induced acute lung injury by improving mitochondrial function. Taken together, our comprehensive analysis indicates that the therapeutic effects of TSC on HALI are generally modest overall. However, TSC significantly reduces ROS contents and effectively mitigates hypoxia-induced histopathological damage in lung tissue.

**Figure 14:**
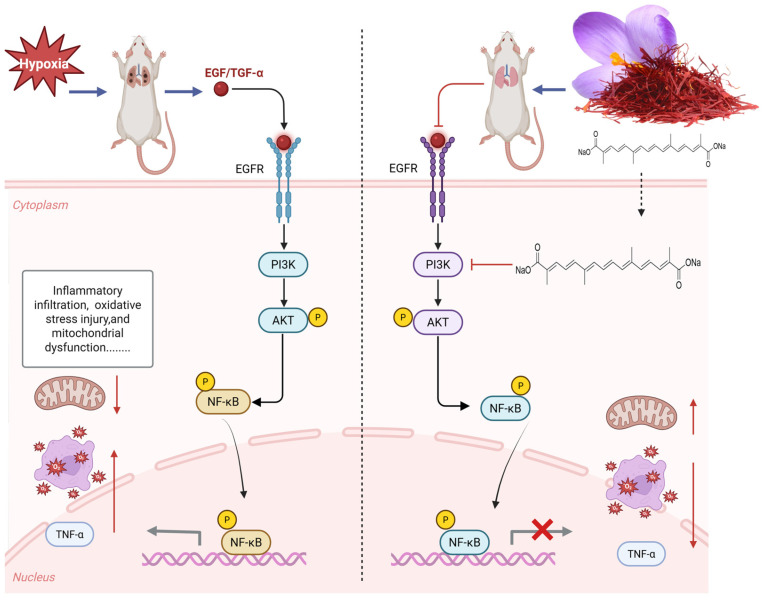
Mechanism of TSC treatment for HALI.

The results of network pharmacology show that TSC mainly acts on 50 targets such as EGFR and AKT1 and exerts its therapeutic effect on HALI by regulating 66 signaling pathways such as PI3K/AKT, reflecting the “multi-target multi-pathway” characteristic of TSC in treating HALI. The epidermal growth factor receptor (EGFR), as a member of the ErbB receptor tyrosine kinase family, plays a crucial role in regulating cell proliferation, survival, and inflammatory response. Recent studies have shown that abnormal activation of EGFR is closely related to the occurrence and development of HALI [24]. Hypoxia can induce alveolar epithelial cells and pulmonary vascular endothelial cells to release ligands such as epidermal growth factor (EGF) and transforming growth factor-α (TGF-α), which activate EGFR through autocrine or paracrine pathways. Activated EGFR promotes the release of pro-inflammatory factors such as TNF-α and MCP-1 through downstream signaling pathways, leading to inflammatory cell infiltration and imbalanced lung tissue repair [25]. Research has shown that the contents of EGFR ligands (such as epidermal growth factor EGF and TGF-α) in bronchoalveolar lavage fluid (BALF) of rats with acute lung injury increase, while the expression of EGFR mRNA in lung tissue is upregulated in a time-dependent manner, leading to increased pulmonary vascular permeability and aggravated neutrophil infiltration, suggesting that EGFR overactivation can exacerbate the pathological process of HALI [25]. The PI3K/AKT/NF-κB signaling pathway is one of the core pathways mediated by EGFR. After EGFR activation, its intracellular tyrosine kinase domain undergoes autophosphorylation, recruiting and phosphorylating the p85 regulatory subunit of phosphatidylinositol 3-kinase (PI3K), which then catalyzes the generation of the second messenger PIP3 and activates AKT (protein kinase B). Activated AKT promotes the phosphorylation of IκBα by the IκB kinase (IKK) complex, promotes NF-κB p65/p50 dimer nuclear translocation, and drives the transcription of various pro-inflammatory factors (such as TNF-α, MCP-1) and chemokines (such as CXCL8), forming a positive feedback loop to exacerbate lung tissue inflammation [26]. During the induction of ALI model in mice by lipopolysaccharide, Zhao et al. [27] found that EGFR, PI3K, AKT, and NF-κB were highly expressed and activated in lung tissue. This study found that acute exposure of rats to simulated high-altitude hypoxia at an altitude of 7000 m significantly increased the expression of EGFR, PI3K, AKT, NF-κB, and mRNA in the lung tissue of hypoxic rats, while TSC could significantly reduce the expression of EGFR, PI3K, AKT, NF-κB, and mRNA. Molecular docking and molecular dynamics simulations also confirmed that TSC could stably bind to EGFR, PI3K, AKT, and NF-κB. The above results indicate that TSC may improve HALI pathological damage by inhibiting the EGFR/PI3K/AKT/NF-κB signaling pathway (Figure 14).

## 5. Conclusions

This article uses network pharmacology combined with in vitro and in vivo experiments to elucidate that TSC downregulates the expression of the EGFR/PI3K/AKT/NF-κB signaling pathway, inhibits inflammatory infiltration and oxidative stress damage, improves mitochondrial function, and thereby alleviates lung tissue damage caused by high-altitude hypoxia. TSC is expected to be developed as a potential drug for treating high-altitude hypoxia-induced lung injury.

## Figures and Tables

**Table 1:** Primer sequence.

Primer Name		Sequence (5′-3′)	Size (bp)
*EGFR*	F	CACCGTGTGGGAACTGATGACC	180
	R	CGGAACTTTGGGCGGCTATC	
*PI3K*	F	CGGGATGCCAAACTCTATGC	109
	R	CACGATGGATGACAATGAAAATG	
*AKT*	F	ATCGTGTGGCAAGATGTGTATGAG	197
	R	GCTGAGTAGGAGAACTGGGGAAA	
*NF-κB*	F	GAGACCTGGAGCAAGCCATTAGC	203
	R	AGTGTTGGGGGCACGGTTATC	
*GAPDH*	F	TTCAGCTCTGGGATGACCTT	129
	R	TGCCACTCAGAAGACTGTGG	

**Table 2:** Binding energy scores and docking parameters of TSC and core target molecules.

Target	Compound	Binding Energy (kcal/mol)
PIK3CA	TSC	−8.5
AKT1	TSC	−7.6
EGFR	TSC	−9.6
IGF-1	TSC	−5.5
NF-κB1	TSC	−6.0

## Data Availability

The data presented in this study are part of an ongoing research project, and sharing preliminary data at this stage could compromise the study’s integrity, but they are available on request from the corresponding author.

## Abbreviations

AKT	Protein kinase B
EGFR	Epidermal growth factor receptor
GSH	Glutathione
HALI	High-altitude acute lung injury
MCP-1	Monocyte chemoattractant protein-1
MDA	Malondialdehyde
NF-κB	Nuclear transcription factor
PI3K	Phosphatidylinositol-3-kinase
SOD	Superoxide dismutase
TSC	Trans-sodium crocetinate
TNF-α	Tumor necrosis factor-α

## References

[1] Wang H., Lin X., Pu X. (2020). NOD-like receptors mediate inflammatory lung injury during plateau hypoxia exposure. J. Physiol. Anthropol..

[2] El Alam S., Pena E., Aguilera D., Siques P., Brito J. (2022). Inflammation in Pulmonary Hypertension and Edema Induced by Hypobaric Hypoxia Exposure. Int. J. Mol. Sci..

[3] Wang R., Ma S., Yang J., Luo K., Qian Q., Pan J., Liang K., Wang Y., Gao Y., Li M. (2024). Sodium Hydrosulfide Protects Rats from Hypobaric-Hypoxia-Induced Acute Lung Injury. Int. J. Mol. Sci..

[4] Ji P., Zhang Z., Mingyao E., Liu Q., Qi H., Hou T., Zhao D., Li X. (2024). Ginsenosides ameliorates high altitude-induced hypoxia injury in lung and kidney tissues by regulating PHD2/HIF-1α/EPO signaling pathway. Front. Pharmacol..

[5] Tang H.J., Wang M.J., Zhao X.J., Lang Y.L., Song D. (2023). Research progress on drug prevention and treatment of high altitude pulmonary edema. Tibet Med. J..

[6] Qian Y.L., Li C., Wang Y., Lin S., Meng Q., Li G., Li J.X., Zhang Y.M. (2025). Research progress on *Crocus sativus* L. and its active components in the treatment of pulmonary diseases. Chin. Herb. Med..

[7] Yang J., Luo K., Guo Z., Wang R., Qian Q., Ma S., Li M., Gao Y. (2024). Evaluation of Crocetin as a Protective Agent in High Altitude Hypoxia-Induced Organ Damage. Pharmaceuticals.

[8] Teng S.S., Hao J., Bi H., Li C., Zhang Y., Zhang Y., Han W., Wang D. (2021). The protection of crocin against ulcerative colitis and colorectal cancer via suppression of NF-κB-mediated inflammation. Front. Pharmacol..

[9] Mehrzadi S., Hosseini P., Mehrabani M., Siahpoosh A., Goudarzi M., Khalili H., Malayeri A. (2021). Attenuation of bleomycin-induced pulmonary fibrosis in wistar rats by combination treatment of two natural phenolic compounds: Quercetin and gallic acid. Nutr. Cancer.

[10] Wu J., Sa Y.P., Zhao X.H., Wang S., Cao N. (2024). Establishment of an acute high altitude hypoxia-induced lung tissue injury model in male rats and exploration of the injury mechanism. Chin. J. High Alt. Med. Biol..

[11] Yuan S.N., Zhang X.Z., Liu J., Fan X., Cao L., Wang Z., Xiao W. (2023). Therapeutic effect of Xingbei Zhike Granules on post-infection cough in guinea pigs. Chin. J. Herb Drugs..

[12] Yang W., Xu H.L., Wu X., Yuan C., Chen S., Luo S., Yuan M., Wang Y., Zhang L., Zhou J. (2024). Effect and mechanism of Shenqi Granules on lipopolysaccharide-induced acute lung injury in mice. Chin. J. Pharmacol. Clin..

[13] Zeng Y., Zhao H., Zhang T., Zhang C., He Y., Du L., Zuo F., Wang W. (2022). Lung-protective effect of Punicalagin on LPS-induced acute lung injury in mice. Biosci. Rep..

[14] Qian Q.Y., Pan J.C., Yang J., Wang R., Luo K., Ma Z., Li M., Gao Y. (2023). Effects of different hypoxia interventions on blood gas and red blood cell-related indices in low-pressure hypoxia model rat. Zhejiang Univ. J. Med. Sci..

[15] Zhao A.P., Ma J.H., Wang Z.H., Chang X., Li W., Wang R. (2023). Protective effect of catechin on acute high altitude hypoxia injury. Chin. J. Pharmacol. Bull..

[16] Pu P., Lu S., Niu Z., Zhang T., Zhao Y., Yang X., Zhao Y., Tang X., Chen Q. (2019). Oxygenation properties and underlying molecular mechanisms of hemoglobins in plateau zokor (*Eospalax baileyi*). Am. J. Physiol. Regul. Integr. Comp. Physiol..

[17] Zhou Y., Huang X., Zhao T., Qiao M., Zhao X., Zhao M., Xu L., Zhao Y., Wu L., Wu K. (2017). Hypoxia augments LPS-induced inflammation and triggers high altitude cerebral edema in mice. Brain Behav. Immun..

[18] Yang X., Li J., Ma Y., Dong X., Qu J., Liang F., Liu J. (2024). Curcumin-mediated enhancement of lung barrier function in rats with high-altitude-associated acute lung injury via inhibition of inflammatory response. Respir. Res..

[19] Hu Y., Sun J., Wang T., Wang H., Zhao C., Wang W., Yan K., Yan X., Sun H. (2021). Compound Danshen Dripping Pill inhibits high altitude-induced hypoxic damage by suppressing oxidative stress and inflammatory responses. Pharm. Biol..

[20] Sharma S., Sandhir R., Ganju L., Kumar B., Singh Y. (2023). Unique mutations in mitochondrial DNA and associated pathways involved in high altitude pulmonary edema susceptibility in Indian lowlanders. J. Biomol. Struct. Dyn..

[21] Tian L., Zhao C., Yan Y., Jia Q., Cui S., Chen H., Li X., Jiang H., Yao Y., He K. (2024). Ceramide-1-phosphate alleviates high-altitude pulmonary edema by stabilizing circadian ARNTL-mediated mitochondrial dynamics. J. Adv. Res..

[22] Huan Y., Quan H., Jia B., Hao G., Shi Z., Zhao T., Yuan Y., Yuan F., Dong Y., Liang G. (2023). High-altitude cerebral hypoxia promotes mitochondrial dysfunction and apoptosis of mouse neurons. Front. Mol. Neurosci..

[23] Li F., Li D., Tang S., Liu J., Yan J., Chen H., Yan X. (2021). Quercetin Protects H9c2 Cardiomyocytes against Oxygen-Glucose Deprivation/Reoxygenation-Induced Oxidative Stress and Mitochondrial Apoptosis by Regulating the ERK1/2/DRP1 Signaling Pathway. Evid. Based Complement Altern. Med..

[24] Lu J., Liu X., Cen A., Hong Y., Wang Y. (2024). HYPOXIA induces lncRNA HOTAIR for recruiting RELA in papillary thyroid cancer cells to upregulate miR-181a and promote angiogenesis. J. Endocrinol. Investig..

[25] Yamaoka T., Arata S., Homma M., Homma T., Kusumoto S., Ando K., Manabe R., Kishino Y., Ohba M., Tsurutani J. (2019). Blockade of EGFR Activation Promotes TNF-Induced Lung Epithelial Cell Apoptosis and Pulmonary Injury. Int. J. Mol. Sci..

[26] Zhou Y., Wang H., Liu A., Pu Z., Ji Q., Xu J., Xu Y., Wang Y., Kumar V. (2024). Sivelestat improves acute lung injury by inhibiting PI3K/AKT/mTOR signaling pathway. PLoS ONE.

[27] Zhao D., Zhang T.Y., Wu Z.Q., Liu G. (2025). Mechanism study of Chaipu Decoction in intervening acute lung injury based on network pharmacology and experimental verification. Chin. J. Inf. Tradit. Chin. Med..

